# Functional immune profiling of hyper- and hypo-inflammatory subphenotypes of critical illness: a secondary analysis

**DOI:** 10.3389/fimmu.2025.1520848

**Published:** 2025-05-09

**Authors:** E. Scott Halstead, Daniel J. McKeone, Abigail M. Samuelsen, Shouhao Zhou, Anthony S. Bonavia

**Affiliations:** ^1^ Division of Critical Care Medicine, Department of Pediatrics, Penn State University College of Medicine, Hershey, PA, United States; ^2^ Department of Molecular and Precision Medicine, Penn State University College of Medicine, Hershey, PA, United States; ^3^ Critical Illness and Sepsis Research Center, Penn State College of Medicine, Hershey, PA, United States; ^4^ Division of Pediatric Hematology/Oncology, Department of Pediatrics, Penn State University College of Medicine, Hershey, PA, United States; ^5^ Division of Critical Care Medicine, Department of Anesthesiology and Perioperative Medicine, Penn State University College of Medicine, Hershey, PA, United States; ^6^ Division of Biostatistics and Bioinformatics, Department of Public Health Sciences, Penn State University College of Medicine, Hershey, PA, United States

**Keywords:** sepsis, biomarker, phenotype, cytokines, critical illness, humans

## Abstract

**Introduction:**

Recent studies of adult sepsis patients demonstrate the existence of two subphenotypes that differ in risk of mortality: a hyper-inflammatory subphenotype with a high risk of mortality, and a hypo-inflammatory or “not hyper-inflamed” subphenotype with a relatively lower risk of mortality. We recently investigated the association of organ dysfunction with *ex vivo* immune profiling in sixty (60) critically ill adult patients with sepsis. In this secondary analysis we measured cytokine biomarkers with an automated, microfluidic immunoassay device (Ella™) and sought to investigate the functional immune profiles of patients in the hyper/hypo-inflammatory subphenotype groups.

**Methods:**

Subjects were consecutively identified adults, older than 18 years, and enrolled within 48 hours of sepsis onset. Whole blood cytokine analysis was performed in all patients. Additionally, *ex vivo* cytokine production was measured following 4h of stimulation. Cytokine concentrations were measured using the Ella™ automated immunoassay system.

**Results:**

Subjects were divided into hypo-inflammatory (42 patients) and hyper-inflammatory (18 patients) subtypes using a previously validated parsimonious model based on concentrations of IL-6, TNFR1 and bicarbonate. The hyper- and hypo-inflammatory clusters demonstrated a near four-fold difference in 30-day mortality (44.4% vs 11.9%, p=0.0046). Following 4h of *ex vivo* stimulation with LPS, TNF production was lower in the hyper-inflammatory group as compared with the hypo-inflammatory group (p=0.0159). *Ex vivo* phorbol 12-myristate 13-acetate (PMA)-stimulated IFN-γ production (4h) by whole blood did not differ between groups.

**Conclusions:**

These data further validate the use of IL-6, TNFR1 and bicarbonate to discern inflammatory sub-groups of patients with critical illness. They also confirm the observation that the presence of the hyper-inflammatory subphenotype is often accompanied by a compensatory anti-inflammatory response syndrome. Future investigations should focus on prospective validation of this panel for prognostic enrichment of clinical research studies.

## Introduction

Sepsis is characterized by dynamic disruptions in the immune system, often leading to an increased susceptibility to secondary infections, long-term health complications, and death (1–5). Biomarkers have shown significant promise in prognostication following acute respiratory distress syndrome (ARDS) (6–10) and sepsis (11, 12): interleukin (IL)-6, IL-8 (otherwise known as C-X-C motif ligand-8 or CXCL8), and tumor necrosis factor receptor 1 (TNFR1). Furthermore, these biomarkers have helped to identify a hyper-inflammatory subphenotype of ARDS and sepsis, characterized by increased mortality as compared with its hypo-inflamed counterpart (10, 13).

Our prior investigation has synthesized existing evidence regarding the prognostic value of whole blood responses to ex vivo lipopolysaccharide (LPS) stimulation of blood in sepsis (14). Reduced TNF production following LPS stimulation remains a hallmark of “immunoparalysis,” a state of immune dysfunction characterized by diminished immune capacity to respond effectively to infections (1, 15, 16). Patients in this state are at a substantially higher risk for developing secondary infections and death (17). However, an exclusive focus on LPS responses may overlook other vital components of immune function, such as lymphocyte count and activity (14, 18).

Both immunoparalysis, indicated by reduced *ex vivo* cytokine production, and hyper-inflammation, characterized by elevated levels of IL-6, IL-8 and TNFR1, are associated with worse outcomes in sepsis. To investigate the apparent paradox between these two states, we examined the relationship between inflammatory subphenotypes as determined by plasma cytokine and bicarbonate concentrations during critical illness, and *ex vivo* cytokine production.

## Materials and methods

### Methodological approach and participant selection

This is a secondary analysis of a recently published study (19). Ethical approval was obtained from the Human Studies Protection Office of the Penn State College of Medicine (#15328, 7/30/2020). Procedures were followed in accordance with ethical standards established by the Human Subjects Protection Office and with the Helsinki Declaration of 1975. The Modified Early Warning Scoring (MEWS) scoring-based algorithm was used to identify critically ill patients with possible sepsis (20, 21) from November 2021 to August 2023. Dual, independent investigator reviews ensured unbiased selection of patients meeting inclusion criteria from electronically flagged patient records. Informed consent was obtained from patients having decision-making capacity, or from the legally authorized healthcare representatives of patients lacking decision-making capacity.

### Inclusion and exclusion parameters

Eligible septic candidates were consecutively identified adults, >18 years, within 48 hours of critical illness onset. Sepsis was defined by Sepsis-3 criteria, namely a change in SOFA score of two or more in the setting of clinically suspected or microbiologically proven infection (22). Non-survivors were defined as those deceased within 30 days post-enrollment. Patients exhibiting active hematologic malignancies and those receiving immune-altering therapies were excluded.

### Clinical variables and data retrieval

Clinical data, including whole blood counts and cell differentials, were retrieved from hospital records and post-discharge interviews. Illness severity was measured using Charlson Comorbidity Index (23) and APACHE II score (24–26). Outcomes included organ dysfunction via SOFA score; 30-day mortality, readmission, and infection rates. Secondary infection was defined using the CDC/NHSN definitions (27). Blood samples were collected early, within 48h of ICU admission, for leukocyte analysis, cytokine analysis, and cytokine quantification after *ex vivo* stimulation tests, with supernatants frozen for later analysis.

### Cytokine analysis

Whole blood was collected in sodium heparin tubes and *ex vivo* cytokine production was performed on the day of collection. Plasma was separated by centrifugation (400 g x 5 min). Plasma was aliquoted and stored at -80°C until subsequent batch analysis. Cytokines were measured using the Ella™ microfluidic automated immunoassay system (Bio-Techne, MN). Biomarkers were measured by batch analysis by Ella™ using custom manufactured 32 sample x 4 analyte cartridges (28–45) (**Supplementary Table 1**). Plasma samples were thawed and diluted 1:10 with sample diluent 13, and 50 µL of diluted sample were loaded into each sample inlet, and data was acquired utilizing the Simple Plex Runner software v.3.7.2.0 (ProteinSimple).

### *Ex vivo* stimulation and cytokine quantification

Whole blood was collected in sodium heparin tubes. As previously described (19) 50 μL whole blood was diluted ten-fold in HEPES-buffered RPMI media, then exposed to designated stimulants (46, 47). Whole blood samples from each study participant were exposed to separate conditions: (1) 500 pg/mL lipopolysaccharide (LPS) from *Salmonella enterica* strain abortus equi, or (2) 10 ng/mL phorbol 12-myristate 13-acetate (PMA) with 1 μg/mL ionomycin. Following incubation for 4 hours at 5% carbon dioxide and 37 °C, and subsequent centrifugation, supernatants were frozen at -80 °C until the time of analysis. The Ella™ automated immunoassay system (Bio-Techne, Minneapolis, MN) was used for triplicate measurement of interferon-gamma (IFN-γ), tumor necrosis factor (TNF), and interleukin (IL)-6. Data was acquired utilizing the Simple Plex Runner software v.3.7.2.0 (ProteinSimple).

### Intracellular cytokine staining

Multicolor flow cytometry was performed on a 17-color Becton Dickinson (BD) FACSSymphony. Blood was obtained from three separate healthy adult controls, ages 40-50, on the morning of stimulation. After 3h of ex vivo stimulation, brefeldin A was added to cause cytokine accumulation in cells. After 4h whole blood cells were surface stained with fluorescent-labeled antibodies (BD or BioLegend) and intracellular staining for TNF and IFN-γ was performed using Cytofix/Cytoperm (BD) kit.

### Statistics

Using Prism (v9.5) and JMP^®^ Pro (v18.0.2), with a significance threshold of 0.05, we summarized variables descriptively. Continuous variables’ distributions were evaluated via histograms, probability plots, and normality tests. Demographic comparisons between groups utilized Chi-square and two-sample t-tests. Due to the non-normal distribution of the cytokine data, log_10_ transformation was applied before modeling. Designation of the inflammatory group (hypo-inflammatory vs. hyper-inflammatory) was determined using a parsimonious mathematical model with IL-6 (pg/mL), TNFR1 (pg/mL) and bicarbonate (mEq/L) values as variables that was previously validated for patients with ARDS (7).

## Results

### Study population and inflammatory clusters

**Figure 1:**
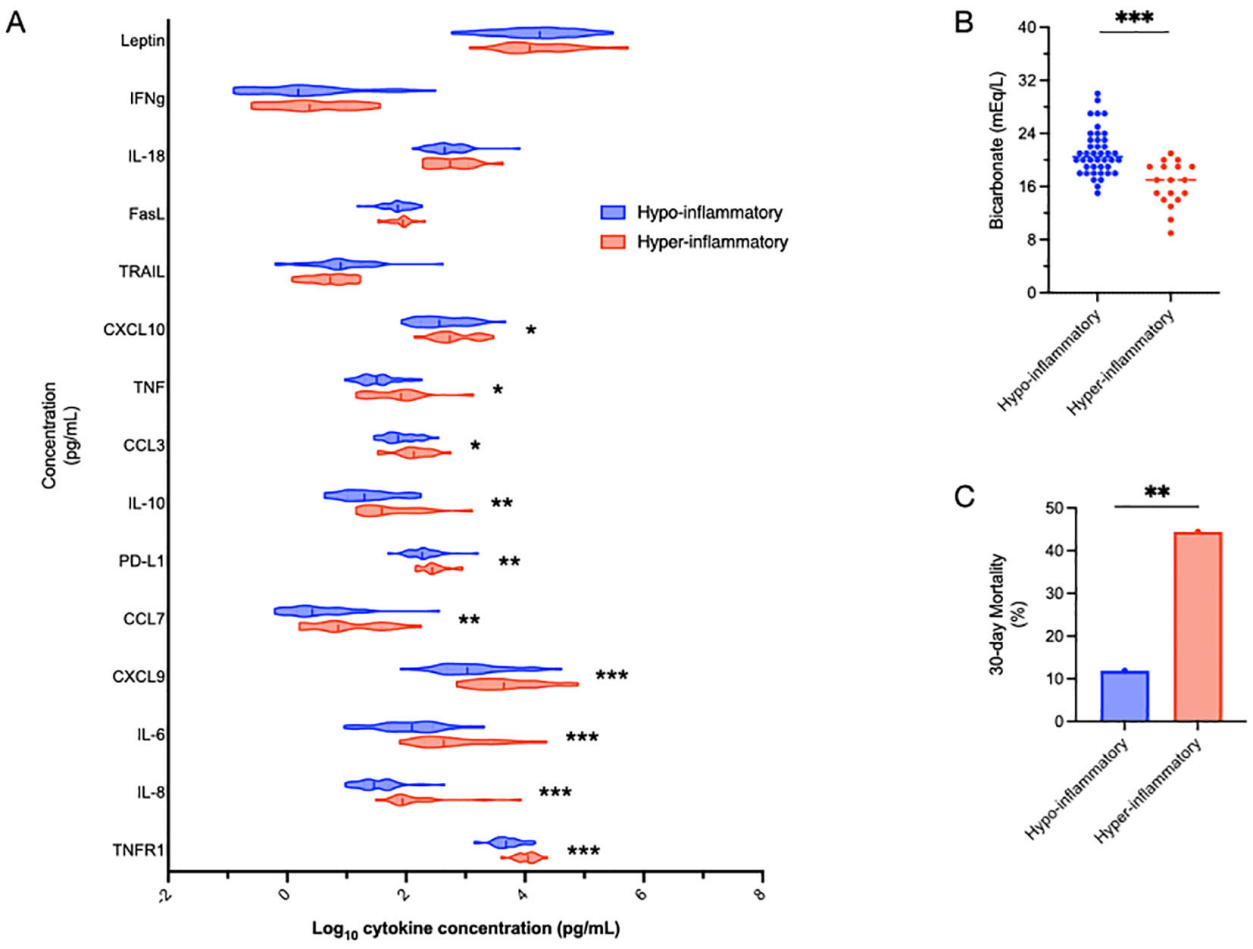
Defining inflammatory subphenotypes by IL-6, TNFR1 and bicarbonate. Sepsis patients were divided into hyper-inflammatory (red) and hypo-inflammatory (blue) groups using IL-6, TNFR1 and bicarbonate levels based on a previously validated model (48). The association of other cytokines with the inflammatory groups is shown, with cytokines listed in order of increasing significant differences between the groups **(A)**. Bicarbonate, the non-cytokine variable in the model, by definition, was significantly different (***p<0.001) between the groups **(B)**. Mortality in the hyper-inflammatory group was significantly higher than the hypo-inflammatory group (44.4% vs 11.9%, **p<0.01) **(C)**. Comparisons between the groups were performed using t-tests **(A, B)** and chi-squared tests **(C)**. *p<0.05.

In the primary study, we enrolled a total of 60 individuals with sepsis. Detailed demographic profiles and clinical outcomes of these patients have been previously described (19). Multiple cytokines were measured in sepsis patients using custom Ella™ cartridges. Using a previously validated parsimonious classifier model (7) based on IL-6, TNFR1 and bicarbonate levels, subjects were divided into two clusters: hyper-inflammatory (defined by elevated IL-6, TNFR1 and low bicarbonate levels, n=18, red), and hypo-inflammatory (n=42, blue) groups (**Figure 1**). The classifier model is a logistic regression-based model comprised of IL-6, sodium bicarbonate and TNFR1. For the model, IL-6 and TNFR1 were log-transformed after the addition of one (+1). The coefficients used for the model were detailed in previous manuscripts (7, 48). We used the validated cutoff of 0.5 for this analysis.

Not surprisingly, TNFR1, IL-8 and IL-6 levels were significantly (***p<0.001) different between the inflammatory subphenotypes (**Figure 1A**). Also not surprisingly, given that bicarbonate is part of the validated model, bicarbonate levels were significantly lower in the hyper-inflammatory cluster (**Figure 1B**). 30-day mortality was almost four-fold higher (p=0.0046) in the hyper-inflammatory (44.4%) group than the hypo-inflammatory (11.9%) cluster (**Figure 1B**).

### Functional immune responses

**Figure 2:**
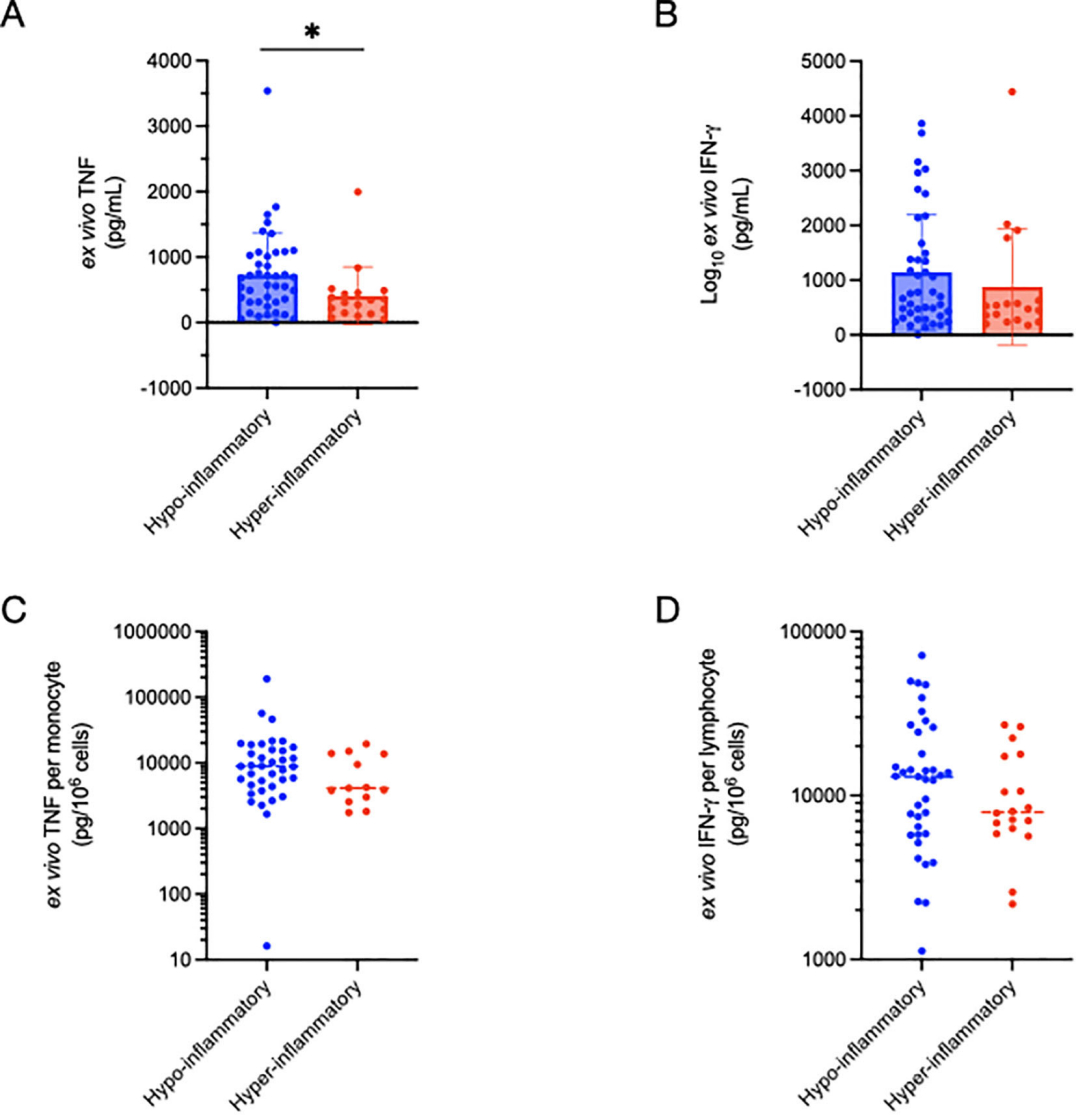
Comparison of functional immune profiling as determined by *ex vivo* cytokine production between inflammatory subphenotypes. Total TNF production (4h) was significantly (*p=0.016) lower in the hyper-inflammatory cluster **(A)**, but not when corrected for absolute monocyte count **(C)**. *Ex vivo* IFN-γ production did not differ between the inflammatory clusters in total **(B)**, or after correction for absolute lymphocyte count **(D)**. Significance was determined using non-parametric (Wilcoxon signed-rank) testing.

We investigated the “functional immune response” by quantifying the amount of cytokines produced by whole blood in response to precise amounts of immune stimulation. We quantified total TNF production after 4h of *ex vivo* stimulation of fresh whole blood with LPS. Interestingly, patients from the hyper-inflammatory cluster demonstrated lower ex vivo TNF production as compared to the hypo-inflammatory group (407±442 vs 729±637 pg/mL, p = 0.0159 (**Figure 2A**). As a side experiment, we investigated what cell types were producing TNF in response to LPS stimulation using intracellular cytokine staining (ICS)(**Supplementary Figure 1**). Patients with sepsis had reduced TNF production as compared to healthy controls (**Supplementary Figures 2**, **4**). While CD15+ neutrophils produced some TNF, by far the largest producers of TNF in the *ex vivo* assay are CD14+ monocytes (**Supplementary Figures 2–4**).

To control for the potential impact of leukopenia on TNF production, we normalized TNF levels to monocyte counts calculated using daily complete blood count with automated differential cell count analysis. TNF production per monocyte (pg TNF/10^6^ cells) did not differ between the inflammatory clusters. In contrast to TNF production in response to LPS stimulation, the total amount of IFN-γ production (pg/mL) following PMA/ionomycin stimulation did not differ between the hyper- and hypo-inflammatory groups (**Figure 2C**), nor was there a difference after normalizing to lymphocyte count (pg IFN-γ/10^6^ cells) (**Figure 2D**).

## Discussion

We have recently described the prognostic value of rapid, functional immune profiling to predict organ dysfunction in critically ill patients (19). This secondary analysis extends our prior investigation by examining the relationship between previously-described, hyper- and hypo-inflammatory subphenotypes of sepsis (11, 12) and *ex vivo* TNF production in our cohort (6–10, 12). By combining measurements of IL-6 and TNFR1 levels in whole blood, combined with bicarbonate levels, with ex vivo production of cytokines in response to stimulation of whole blood, we observed that hyper-inflamed patients produce less TNF following *ex vivo* LPS stimulation, as compared with hypo-inflammatory sepsis patients. *Ex vivo* TNF production by whole blood has typically been ascribed to monocytes (49). We were able to confirm this using intracellular cytokine staining (**Supplementary Figures 1–4**). The white blood cell and absolute monocyte counts were not different between the inflammatory subphenotypes. Therefore, we expected to see less TNF production per monocyte in the hyper-inflammatory sub-group, but this result was not significant (p=0.138, Wilcoxon two-sample test). This is likely due to our sample size being underpowered to detect the difference. Furthermore, the AMC was based on automated differential counts from the clinical laboratory which can be fairly inaccurate (50).

These results support the existing paradigm of a concurrent systemic inflammatory (SIRS) and compensatory anti-inflammatory (CARS) response in acute sepsis (15, 51). In our cohort, being in the hyper-inflammatory subgroup increased the 30-day risk of mortality nearly 4-fold. Further research needs to be performed to develop strategies rapidly identify this group, and to provide anti-inflammatory interventions. The ongoing NICHD supported PRECISE trial (52) is examining the role of IL-1 blockade using anakinra to decrease organ dysfunction. It is also plausible that targeted anti-inflammatory agents may restore *ex vivo* TNF production.

Our work adds to a growing body of literature that demonstrates the value of IL-6, IL-8 and TNFR1 concentrations for prognostic enrichment of clinical cohorts. IL-6 and IL-8 are very tightly correlated: R^2^ 0.95 in our cohort of sepsis patients. While IL-6 is described as one of the big three pyrogens (IL-1, IL-6, TNF), IL-1 and TNF seem to be the main drivers of inflammation, while IL-6 serves as a quality biomarker (53). IL-8, more correctly named CXCL8 (54), is a chemokine secreted by toll-like receptor-expressing cells. It binds to CXCR1 with a higher affinity as compared with CXCR2, and it functions as neutrophil chemoattractant (55). TNFR1 is one of two TNF receptors that are cleaved from cell surfaces following TNF signaling. TNFR1 is ubiquitously expressed, whereas the expression of TNFR2 is restricted to immune cells (56, 57). The use of TNFR1 as a prognostic biomarker of mortality in critical illness is well established. Elevated TNFR1 levels likely reflect both prior and ongoing TNF signaling following a pro-inflammatory insult (58).

The role of the ex vivo TNF production assay for clinical use is unclear (14). In the present study ex vivo TNF did not predict 30-day mortality. The assay is likely a better predictor of risk of secondary infection, but this study was underpowered to detect any association. While the ex vivo TNF production assay is relatively easy to perform, it is time intensive and requires a 4h incubation step. Also, comparisons of the assay results across laboratories is not standardized.

The methods for conducting *ex vivo* LPS-stimulated TNF production assays vary across laboratories and investigators (14). Our laboratory utilizes the same LPS source (*Salmonella enterica*) and dose (500 pg/mL) as Dr. Hall from Nationwide Children’s Hospital (15, 16). As a result, our TNF measurements may differ from those obtained by other sepsis research laboratories. The same limitation is also true of the PMA/ionomycin-induced IFN-γ assay.

The chief limitation of the present study is the relatively small sample size, which likely limited our ability to detect some significant differences between the two subphenotypes.

Strengths of our study include the use of a rapid, established and semi-automated immunoassay system for cytokine measurement directly from plasma, and of supernatants for the ex vivo stimulation assays. Because of the Ella™ system’s attributes, which include high sensitivity, a large dynamic measurement range, rapid analysis, and high reproducibility (19, 46, 47), we envision the proliferation of its use as a clinical instrument for on-site cytokine measurements in the near future. Future studies should focus on the rapid differentiation of the two subphenotypes with targeted interventions.

## Data Availability

The raw data supporting the conclusions of this article will be made available by the authors, without undue reservation.

## Supplementary material

Supplementary Table 1 Biomarker selection.
