# Supplementary material for: Functional immune profiling of hyper- and hypo-inflammatory subphenotypes of critical illness: a secondary analysis
Source: Front Immunol. 2025 May 9;16:1520848. doi: 10.3389/fimmu.2025.1520848 (PMC12109463; doi:10.3389/fimmu.2025.1520848)
Supplement: Supplementary Table 1 — Biomarker selection. [file Table1.docx]

| Supplemental Table 1: Biomarker selection | | |
| --- | --- | --- |
| Biomarker | Reason for inclusion | References |
| CCL3 | Chemokine, prognostic marker of mortality in children (sepsis) | (41, 42) |
| CCL7 | Chemokine, prognostic marker of mortality in children (ARDS) | (43-45) |
| CXCL9 | Chemokine Marker of IFN activity, specifically IFN-γ | (46, 47) |
| CXCL10 | Chemokine marker, marker of IFN activity | (48, 49) |
| FasL | TNF receptor family member, ligand for Fas | (50) |
| IFN-γ | Type II interferon | (46) |
| IL-6 | Cytokine, prognostic marker of mortality | (51) (52) |
| IL-8 | Chemokine Prognostic marker of mortality | (11) |
| IL-10 | Cytokine, anti-inflammatory | (50) |
| IL-18 | IL-1 family cytokine, associated with autoimmune processes | (53) |
| Leptin | Adipokine | (54) |
| PD-L1 | Ligand for PD-1, checkpoint molecule | (55) |
| TNF | Cytokine | (56) |
| TNFR1 | Prognostic marker of mortality | (38) (11) |
| TRAIL | Distinguish viral from bacterial infections | (57) (58) |
