# Supplementary figures and images for: Functional immune profiling of hyper- and hypo-inflammatory subphenotypes of critical illness: a secondary analysis
Source: Front Immunol. 2025 May 9;16:1520848. doi: 10.3389/fimmu.2025.1520848 (PMC12109463; doi:10.3389/fimmu.2025.1520848)

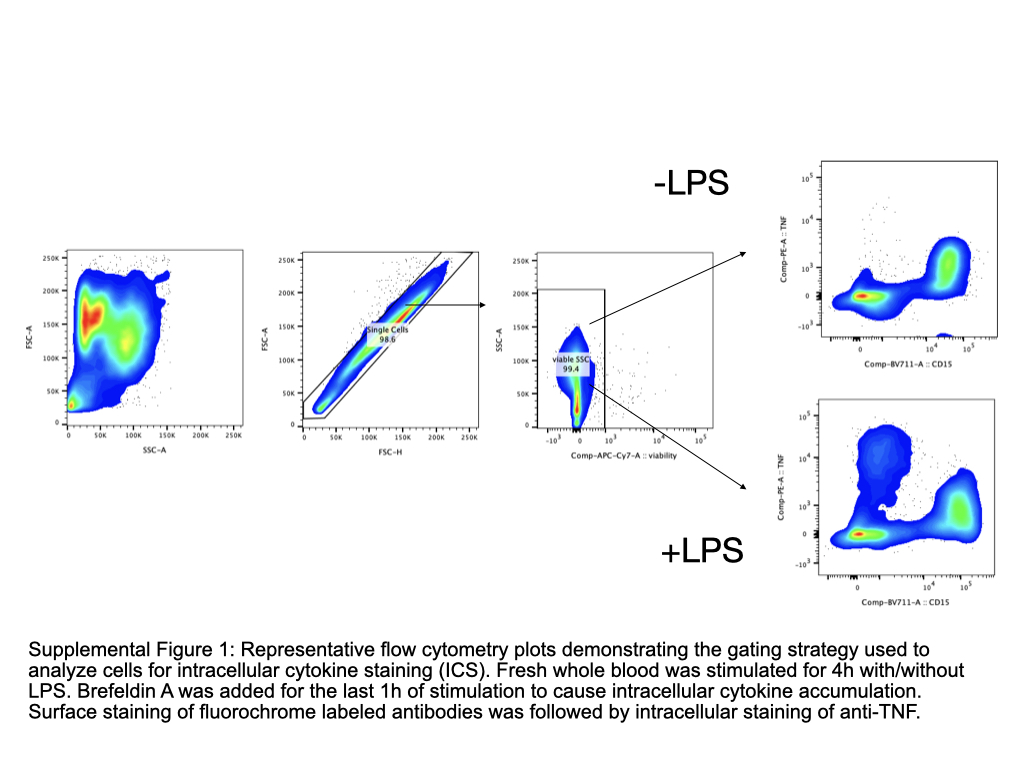

Supplement: Supplementary file 2 [file Image1.jpeg]

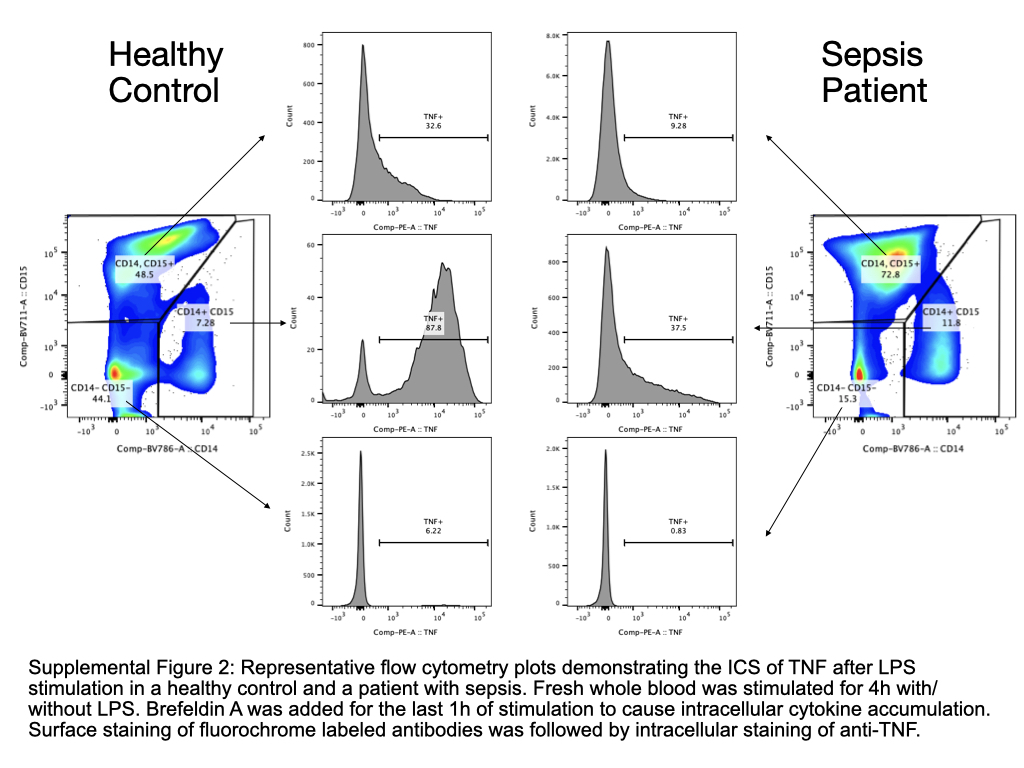

Supplement: Supplementary file 3 [file Image2.jpeg]

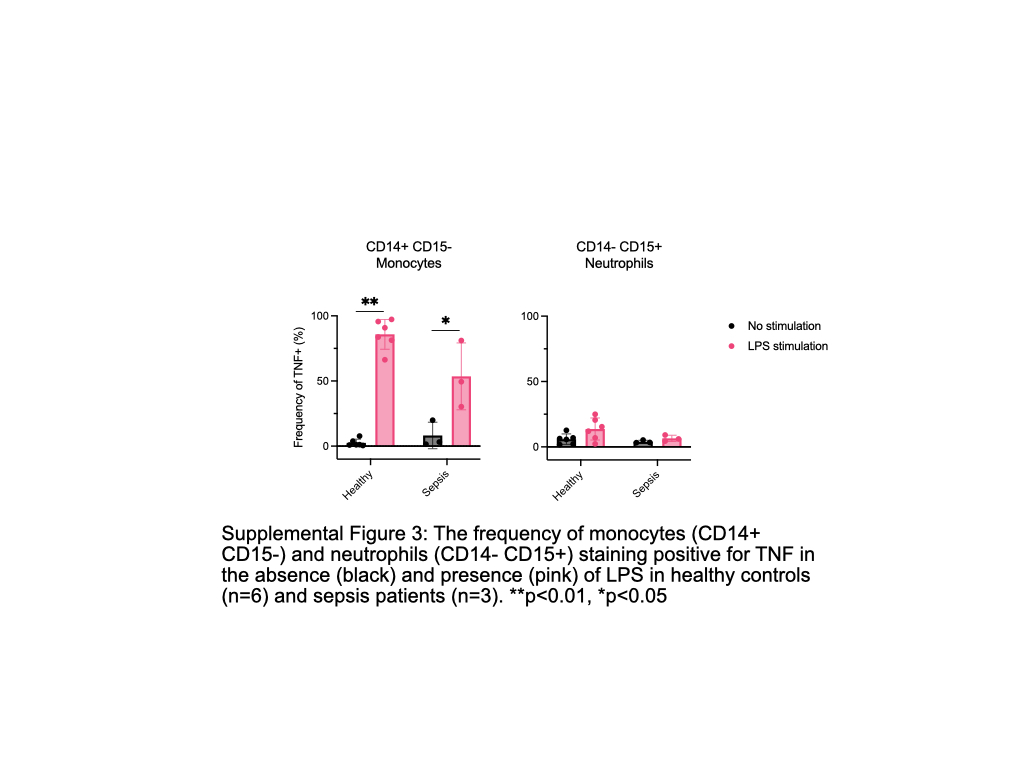

Supplement: Supplementary file 4 [file Image3.jpeg]

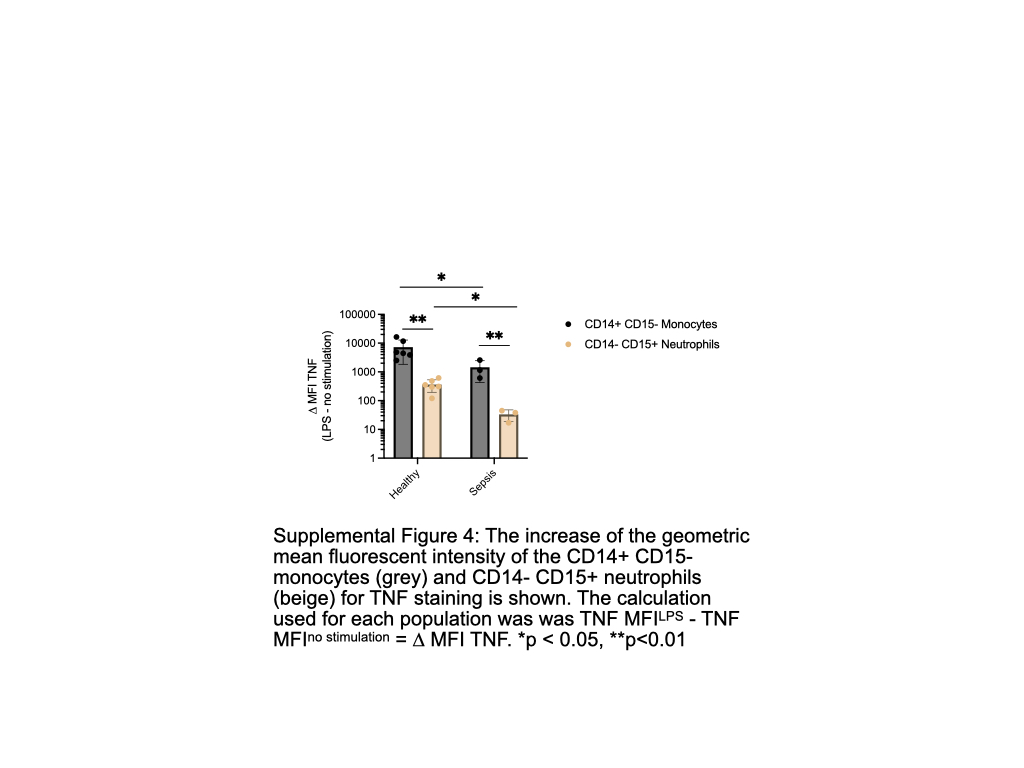

Supplement: Supplementary file 5 [file Image4.jpeg]
